# Migration of Bisphenol A (BPA) into PET-Bottled Beverages: A Comprehensive Review and Bibliometric Analysis

**DOI:** 10.3390/toxics14090796

**Published:** 2026-09-09

**Authors:** Tonny Briceño, Grobert A. Guadalupe

**Affiliations:** Grupo de Investigación en Seguridad Alimentaria (GISA), Instituto de Investigación, Innovación y Desarrollo para el Sector Agrario y Agroindustrial (IIDAA), Universidad Nacional Toribio Rodríguez de Mendoza de Amazonas, Calle Higos Urco N° 342-350-356/Calle Universitaria N° 304, Chachapoyas 01001, Peru; 7414008122@untrm.edu.pe

**Keywords:** food contact materials, endocrine disruptors, bottled beverages, polyethylene terephthalate (PET), chemical migration, food safety, risk assessment

## Abstract

The presence of bisphenol A (BPA) in beverages packaged in polyethylene terephthalate (PET) has attracted increasing attention due to its endocrine-disrupting effects and the controversy over its origin, since virgin PET does not use BPA as a monomer. This review aimed to analyze evidence on BPA occurrence in PET-packaged beverages, migration from PET itself, associated factors, analytical methodologies, and potential contamination sources. A literature search was conducted in Scopus following PRISMA guidelines, resulting in 60 studies analyzed using Bibliometrix (Biblioshiny), VOSviewer, and qualitative evidence synthesis. Publications increased markedly after 2020, with a thematic shift toward food safety, contaminant migration, and circular economy. Temperature, storage time, ultraviolet radiation, beverage characteristics, packaging composition, and recycled PET were identified as factors associated with BPA occurrence or migration. However, the available evidence does not conclusively demonstrate that BPA detected in PET-packaged beverages originates from the migration of virgin PET. Moreover, substantial heterogeneity in analytical methods, detection limits, sample matrices, and experimental conditions limits direct comparison of BPA concentrations and the strength of overall conclusions. Standardized analytical approaches and robust studies are needed to clarify BPA sources and support future risk assessment and regulatory decisions.

## 1. Introduction

The use of polymeric materials in the food industry has increased steadily over the past decades owing to their technological, economic, and functional advantages, including low weight, mechanical strength, transparency, and ease of processing [1]. Among these materials, polyethylene terephthalate (PET) has become one of the most widely used packaging materials for beverages, including bottled water, soft drinks, energy drinks, fruit juices, and dairy products, due to its ability to preserve product quality while facilitating large-scale distribution and commercialization [2,3]. However, the prolonged contact between food products and polymeric packaging materials has raised increasing scientific and public health concerns regarding the potential migration of chemical compounds from packaging into food and beverages.

Among these compounds, bisphenol A is one of the most extensively investigated emerging contaminants. BPA is a synthetic organic compound primarily used in the manufacture of polycarbonate plastics and epoxy resins employed in food packaging, internal can coatings, and other food-contact materials. According to the European Food Safety Authority (EFSA), human exposure to BPA remains a significant concern due to its potential to migrate into food matrices under specific conditions of use and storage, particularly at elevated temperatures, under ultraviolet radiation, and during prolonged storage [4].

The concern surrounding BPA primarily relates to its activity as an endocrine-disrupting chemical (EDC) [5]. Numerous studies have demonstrated its ability to interfere with endocrine function through estrogenic mechanisms and have reported associations with metabolic, reproductive, immunological, and neurological alterations [6,7,8]. In addition, growing evidence suggests potential links between chronic low-dose BPA exposure and carcinogenic processes, further increasing scientific and regulatory interest in its occurrence in food-contact materials [9,10,11,12].

Although BPA is intentionally used in the production of polycarbonate plastics and epoxy resins, numerous recent studies have reported its presence in beverages packaged in PET containers, giving rise to an unresolved scientific controversy. From a chemical perspective, virgin PET does not use BPA as a structural monomer during its conventional synthesis. Nevertheless, experimental studies have consistently detected BPA in beverages stored in PET packaging, often at concentrations that vary with specific storage and handling conditions [13,14,15,16]. This apparent contradiction has led to several hypotheses regarding the origin of BPA, including contamination from recycled PET (rPET), cross-contamination during manufacturing, the presence of non-intentionally added substances (NIAS), other packaging components, and polymer degradation induced by environmental factors [17].

In this context, a comprehensive systematic review is needed to critically integrate the available scientific evidence, compare the experimental findings reported in the literature, and examine the existing controversies regarding the occurrence and migration of BPA in PET beverage packaging. Therefore, the aim of the present systematic review was to analyze the scientific evidence on the occurrence, migration, and toxicological relevance of BPA in PET-packaged beverages, identifying the factors influencing this phenomenon and the potential sources of BPA reported in the scientific literature.

## 2. Search Methodology

### 2.1. Literature Search and Selection

**Figure 1:**
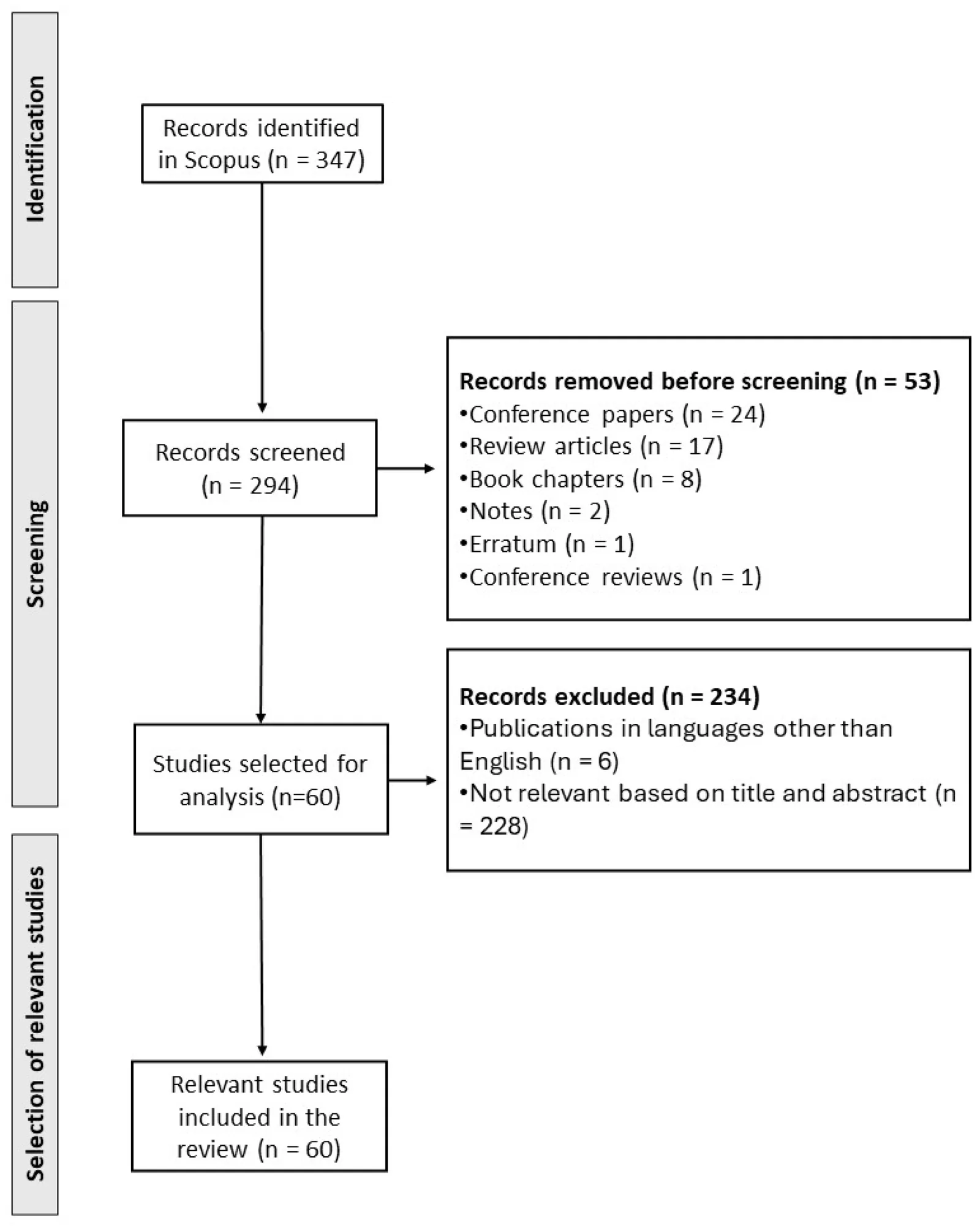
PRISMA flow diagram illustrating the systematic literature search and study selection process (search conducted on 20 May 2026).

The Preferred Reporting Items for Systematic Reviews and Meta-Analyses (PRISMA) framework was used to conduct a comprehensive systematic search of previously published studies on the migration of BPA from beverages packaged in polyethylene terephthalate (PET) [18]. The Scopus database was selected as the primary source of publications because of its broad coverage of scientific literature, high indexing standards, and availability of specialized tools for bibliometric and literature analyses. The literature search was performed using the following query: (“Bisphenol A” OR “BPA”) AND (“polyethylene terephthalate” OR “PET”). The search was conducted across article titles, abstracts, and keywords. Subsequently, all retrieved records were screened to confirm their relevance to the study objective, as illustrated in Figure 1.

A total of 347 records were initially identified in the Scopus database using the predefined search strategy for the migration of BPA from PET packaging. During the screening stage, automated inclusion and exclusion criteria were applied using the Bibliometrix package, resulting in the removal of 53 records that did not correspond to primary research articles, including conference papers (n = 24), review articles (n = 17), book chapters (n = 8), notes (n = 2), errata (n = 1), and conference reviews (n = 1), leaving 294 records for further evaluation. During the eligibility stage, studies published in languages other than English (n = 6) were excluded. The remaining 288 records were subsequently assessed through a detailed evaluation of titles, abstracts, and full texts to ensure thematic relevance, and studies that did not directly address the occurrence and migration of BPA in beverages packaged in PET were excluded. Ultimately, 60 studies met all eligibility criteria and were included in the systematic review and bibliometric analysis.

### 2.2. Bibliometric Analysis

A systematic literature review, complemented by bibliometric analysis and data mining, was conducted to identify the main trends, methodologies, and findings regarding the presence and migration of BPA in beverages packaged in PET containers. The bibliographic search was conducted in Scopus using a structured search strategy based on terms related to bisphenol A, PET packaging, and migration processes. The retrieved records were exported in CSV format for subsequent processing and analysis. The bibliographic information was processed using RStudio (version 2026.08.2) and the Bibliometrix package (version 5.5.0) via its interactive web interface, Biblioshiny, which enabled descriptive analyses of scientific output, publication trends, keyword analysis, and evaluation of the literature’s conceptual structure through co-occurrence networks. Additionally, VOSviewer software (version 1.6.21) was used to construct and visualize bibliometric maps based on relationships among keywords, terms, and thematic clusters, thereby identifying research trends and connections among topics associated with BPA migration from PET packaging into beverages. After the identification, screening, and selection of studies according to the established eligibility criteria, a systematic extraction of experimental information was performed, including variables such as beverage type, PET container characteristics, storage conditions, temperature, exposure time, migration factors, analytical techniques for BPA determination, and reported BPA concentrations. Finally, the information obtained from the bibliometric analysis and the detailed assessment of the selected studies was synthesized through comparative tables, graphical representations, and network maps, facilitating the interpretation of factors influencing BPA migration and current research trends in food safety.

## 3. Results and Discussion

### 3.1. Performance and Network Analysis

#### 3.1.1. Annual Scientific Production

**Figure 2:**
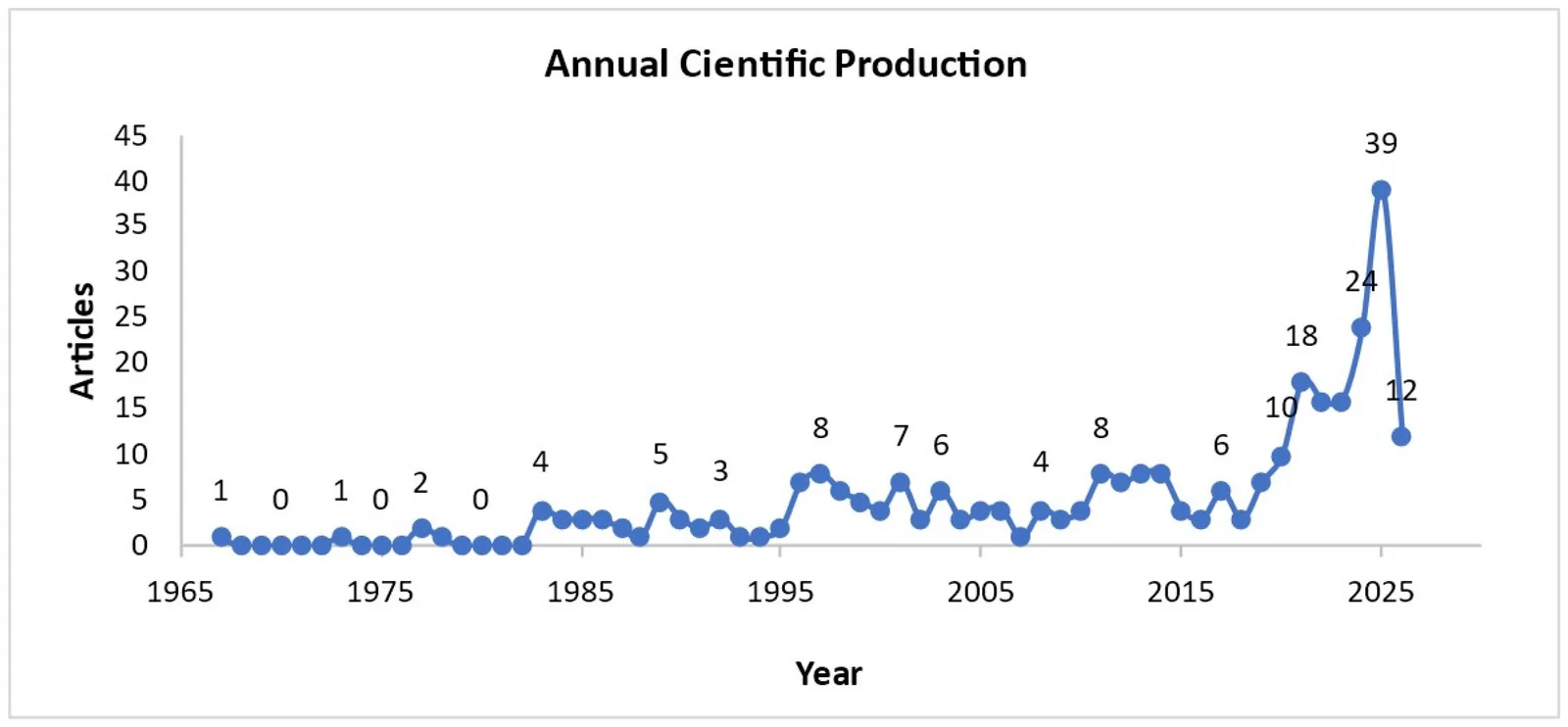
Annual scientific production related to BPA migration in PET-packaged beverages from 1967 to 2026.

Research on the presence of BPA in PET packaging dates back to 1967, when the first publication on this topic was published. During the following decades, between 1967 and 1990, scientific activity remained limited and sporadic, with only one or a few publications per year. From 1991 to 2019, scientific output increased gradually, with moderate fluctuations and relatively stable productivity. However, from 2020 onwards, research activity experienced a more pronounced growth, reaching 15 publications per year between 2021 and 2023, followed by an increase to 21 articles in 2024. The trend peaked in 2025, with 36 publications, demonstrating growing interest within the scientific community in the potential presence of BPA in PET-based systems. This increase may be associated with growing concerns regarding BPA as an endocrine-disrupting compound, the strengthening of regulations on food contact materials, the reassessment of exposure limits by international organizations, and the increasing interest in the safety of plastic packaging and the use of recycled PET within current sustainability and circular economy approaches, as shown in Figure 2.

#### 3.1.2. Scientific Production by Country

**Figure 3:**
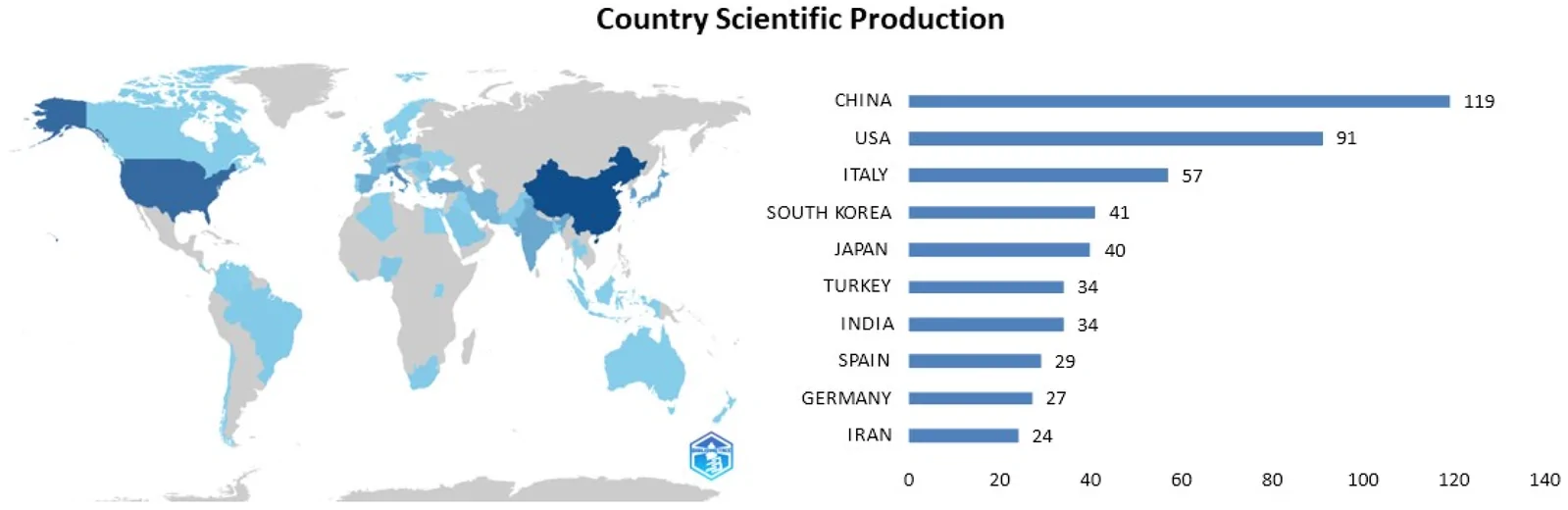
Scientific production by country and top global contributors in bisphenol A research in PET.

Research on the presence of bisphenol A in PET packaging has been developed worldwide. Leading in scientific production, in terms of the number of publications, are China (119 publications), the United States (91 publications), and Italy (57 publications). Likewise, Figure 3 provides a visual representation of the geographical distribution of scientific production, where color intensity indicates the volume of publications by country, highlighting a high concentration of research activity in East Asia, North America, and Western Europe. Additionally, the graph presents the top 10 leading countries in scientific production on this topic.

#### 3.1.3. Contribution of the Journals

**Figure 4:**
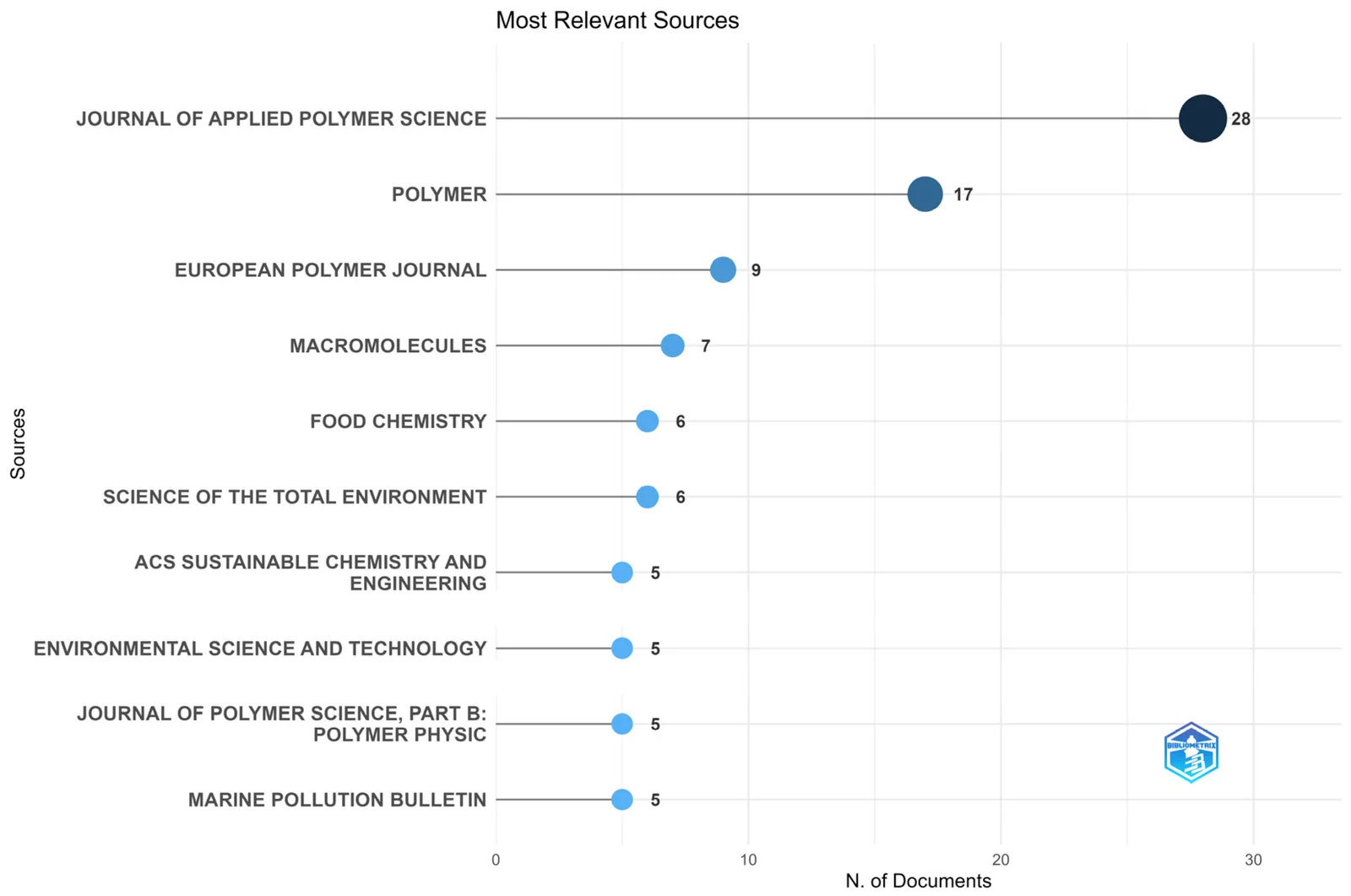
Distribution of publications by leading scientific journals on the topic.

Journals were evaluated based on the number of publications and their scientific impact in the research area. Among the 296 documents retrieved from Scopus, 165 sources contributed publications; however, only 11 journals published 5 or more articles. Figure 4 presents the most significant journals and their publication counts on this topic, reflecting a high concentration of research in journals specialized in polymer science, food chemistry, and environmental safety. The Journal of Applied Polymer Science leads in scientific output with 28 articles, followed by Polymer with 17 and European Polymer Journal with 9. Other highly relevant sources include Macromolecules (7 articles), Food Chemistry, and Science of the Total Environment, with 6 publications each.

#### 3.1.4. Contribution of the Authors

Mapping the most influential research agents enables identification of the scientific front and the concentration of intellectual capital in the field of chemical safety of packaging materials. Bibliometric activity indicators position Berti, C., at the forefront of indexed scientific production, with 6 contributions, followed closely by Eguiazábal, J., and Fiorini, M., each reporting 5 manuscripts. This group of prominent authors represents the cognitive foundation of the analyzed dataset, guiding academic trends toward the study of barrier properties, polymer behavior in food-contact applications, and the factors that determine contaminant mass transfer.

The complete distribution of the most productive authors is presented in Figure S1, which illustrates the publication output of the leading contributors and highlights the concentration of scientific production among a limited number of researchers.

Scientific collaboration, or co-authorship, is essential for the advancement of research. Among the 288 articles analyzed using Biblioshiny, only 12 were authored by a single author, indicating that individual research is uncommon in this field. In contrast, collaborative research was highly predominant, with an average of 4.8 authors per document. This high rate of co-authorship highlights the multidisciplinary efforts required to address the complexities of chemical migration and food safety regulations.

#### 3.1.5. Contribution of the Affiliations

The institutional analysis enables the identification of research centers and academic networks that lead scientific production on the presence and migration of bisphenol A in PET packaging. Based on the analyzed dataset, a broad international network of affiliations was identified, in which a selected group of institutions accounts for the highest frequency of publications. The University of Missouri (United States) leads in scientific output with 12 articles, followed by the University of Science and Technology of China (China) with 6 publications. Likewise, notable contributions were reported from Recep Tayyip Erdogan University (Türkiye) and the University of Liberia (Liberia), with 5 articles each. The presence of these institutions at the forefront reflects the global and multidisciplinary effort devoted to studying the safety of food contact materials and evaluating associated toxicological risks.

Figure S2 presents the complete ranking of the most productive affiliations in the bibliometric dataset, showing the distribution of publications across leading institutions and their relative contributions to the research field.

#### 3.1.6. Keyword Analysis

**Figure 5:**
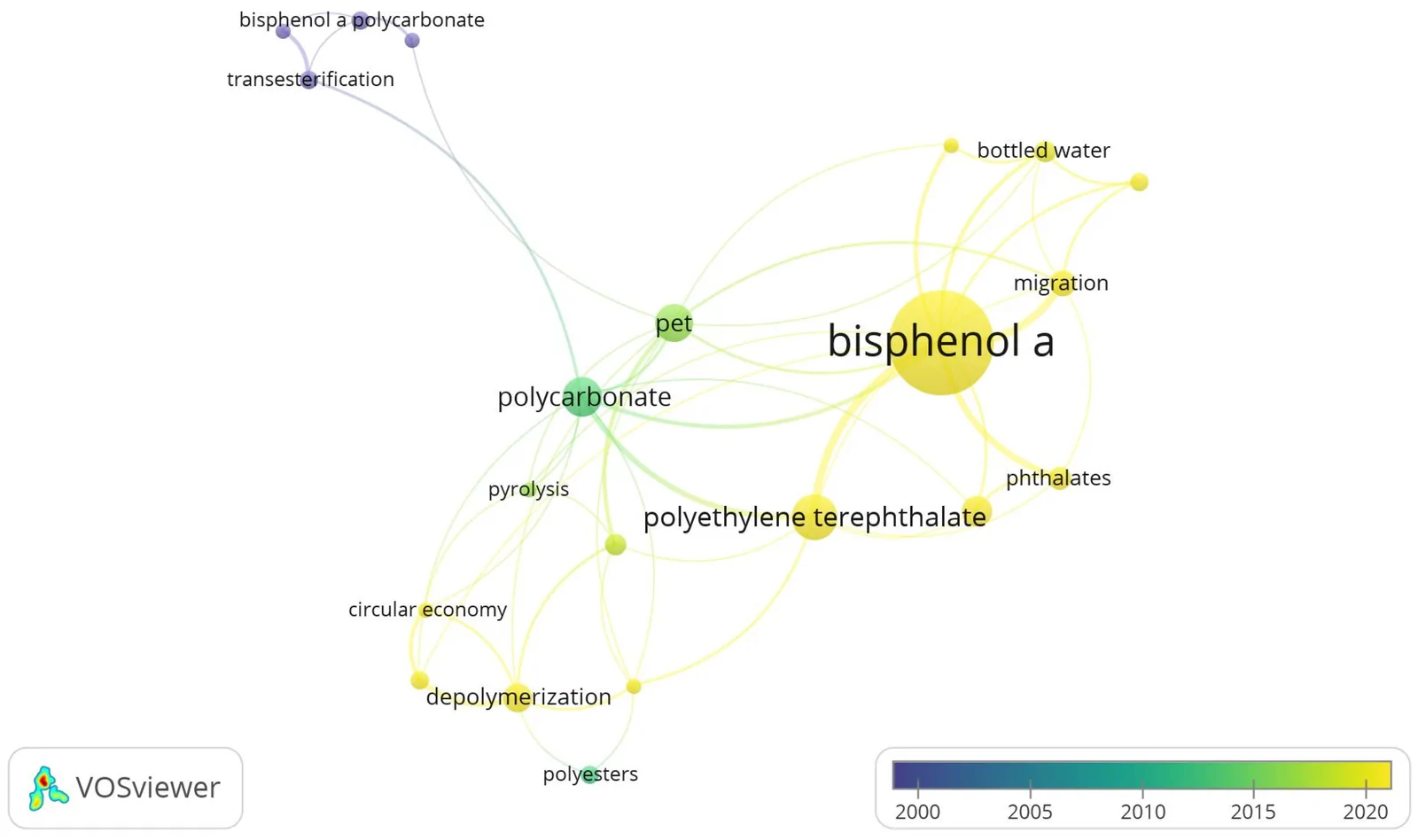
Overlay visualization of the bibliometric map of highly relevant terms and research trends associated with bisphenol A.

Figure 5 shows the Overlay visualization of the keyword co-occurrence network obtained using VOSviewer, where node size represents term frequency, links indicate their degree of association, and the color scale reflects the average publication year (blue: older studies; yellow: more recent studies). The term “bisphenol A” was identified as the central and most frequent node, being closely related to “migration”, “bottled water”, “polyethylene terephthalate (PET)”, and “phthalates”. This indicates that recent research has focused on BPA migration from packaging materials into food and beverages, particularly bottled water. Likewise, “polycarbonate” and “PET” act as connecting nodes between studies on polymeric materials and migration processes, whereas terms such as “transesterification” and “bisphenol A polycarbonate”, represented in blue-violet tones, correspond to earlier research lines focused on polymer chemistry and transformation processes. In contrast, keywords such as “migration”, “bottled water”, “depolymerization”, and “circular economy”, identified in yellow tones, reflect an evolution toward research focused on food safety, consumer exposure, and the sustainability of plastic materials. These findings demonstrate a shift from a primarily chemical perspective toward applications related to health risk assessment and plastic packaging management.

### 3.2. BPA in PET: Factors Influencing Its Migration and Potential Sources of Contamination

The detection of BPA in beverages stored in PET containers cannot be attributed to a single mechanism but rather to the interaction of multiple factors, including storage conditions, food matrix characteristics, and the packaging system itself [19]. Overall, temperature is consistently recognized as one of the most influential variables affecting BPA concentrations in PET-packaged beverages [20]. Exposure of PET bottles to temperatures above normal ambient conditions, particularly during prolonged storage, has been associated with increased BPA concentrations in the beverage. This behavior has been attributed to enhanced molecular mobility and accelerated diffusion processes of compounds present within the packaging system. However, the magnitude of this effect varies considerably among studies. While some investigations reported progressive increases in BPA concentration with increasing temperature, others observed only minor changes or concentrations remaining below the analytical limit of detection. These discrepancies suggest that BPA occurrence is not solely governed by temperature but by the combined influence of multiple interacting factors.

Storage time has also been identified as an important factor influencing BPA occurrence [21]. The general trend indicates that prolonged contact between the packaging material and the beverage favors the appearance or increase of BPA traces [22,23]. This effect becomes particularly evident when samples are simultaneously subjected to unfavorable storage conditions, such as elevated temperatures or direct solar radiation. Nevertheless, the relationship between storage duration and BPA concentration is not always linear. Several studies reported relatively stable concentrations after specific storage periods, whereas others observed fluctuations attributable to experimental variability and the analytical limitations inherent in quantifying compounds present at extremely low concentrations [24,25].

Light exposure, particularly ultraviolet (UV) radiation, has also been extensively investigated as a potential factor influencing BPA occurrence. Photooxidation and material aging may modify the surface structure of PET containers, potentially promoting the release of substances associated with the packaging system [26]. Although most studies agree that UV radiation affects the stability of plastic materials, findings on BPA remain inconsistent, highlighting the complexity of the underlying mechanisms and suggesting that components other than PET itself may contribute to BPA detection.

The characteristics of the food matrix also play an important role in the behavior of chemical contaminants. Variables such as pH, carbon dioxide (CO_2_) content, alcohol concentration, and overall chemical composition may influence the extraction and solubilization of specific compounds [27,28,29]. Accordingly, several studies have reported higher BPA concentrations in beverages with particular physicochemical characteristics, whereas others found no significant differences among beverage types [30,31,32,33]. These inconsistencies indicate that BPA migration from packaging materials is governed by the combined influence of multiple factors rather than by the nature of the beverage alone. Indeed, BPA has also been detected in relatively neutral matrices such as bottled water, suggesting that packaging characteristics, storage conditions, and environmental exposure may substantially contribute to its occurrence [34,35,36].

Another factor frequently highlighted in the literature is the manufacturing process and composition of the packaging system. The incorporation of recycled PET, differences among commercial brands, raw material quality, blow-molding and thermoforming processes, as well as the characteristics of caps, seals, and other packaging components, have all been identified as potential sources of variability among studies [37,38]. Considerable differences in BPA concentrations have been reported even among apparently similar PET containers, suggesting that manufacturing conditions may significantly influence the occurrence of contaminants within the overall packaging system.

Finally, a substantial proportion of the variability reported across studies appears to be associated with the analytical methodologies employed [39,40]. Differences in extraction procedures, sample preparation protocols, limits of detection (LOD), and the instrumental sensitivity of analytical techniques such as high-performance liquid chromatography (HPLC), liquid chromatography–tandem mass spectrometry (LC–MS/MS), and gas chromatography–mass spectrometry (GC–MS) may lead to considerable variation in the reported BPA concentrations. Consequently, direct comparison of results among studies should be interpreted with caution, taking into account the methodological characteristics of each investigation [41,42,43]. To facilitate this comparison, Table 1 summarizes the principal analytical characteristics of the studies included in this review, including the analytical techniques employed, detection and quantification limits, and the BPA concentrations reported.

### 3.3. Potential Sources of Contamination Associated with BPA Detection in PET Packaging Systems

Although PET does not use bisphenol as a chemical precursor during its synthesis, numerous studies have reported the presence of this compound in beverages packaged in PET containers, sparking considerable debate about the actual source of the contamination [54,55]. An initial interpretation could attribute this phenomenon to the packaging material itself, as BPA concentrations tend to increase under adverse storage conditions, including elevated temperatures, prolonged exposure to ultraviolet (UV) radiation, and extended storage periods [56,57]. However, BPA has also been detected in beverages packaged in chemically inert materials such as glass, in some cases at concentrations even higher than those reported for PET containers [51,58]. This evidence suggests that the packaging material alone cannot fully account for the presence of BPA and supports the hypothesis of a multifactorial origin involving different stages of the production and distribution chain.

One of the most widely accepted hypotheses involves the use of recycled PET (rPET). The increasing incorporation of recycled materials, driven by circular economy strategies, has raised the likelihood of cross-contamination during the collection, sorting, and recycling of plastic waste [57,59]. During these processes, accidental mixing with polycarbonate plastics and epoxy resins, both of which contain BPA, may occur, allowing trace amounts of the compound to remain in recycled materials and subsequently be incorporated into newly manufactured PET containers [55]. Although this hypothesis is chemically plausible, the available evidence does not yet permit precise quantification of rPET’s contribution to the BPA concentrations detected in commercially packaged beverages.

Another potential source involves secondary components of the packaging system. While most studies have focused on the PET bottle itself, the packaging system also includes caps, seals, adhesives, labels, printing inks, and various coatings whose chemical compositions differ from that of PET [60]. Some of these materials may contain phenolic compounds or substances structurally related to BPA that can migrate into food or beverages under specific storage conditions. Therefore, the evaluation of BPA migration should not be limited to the primary polymer but should encompass all materials that comprise the packaging system.

Contamination may also occur during manufacturing and packaging operations. Several studies have suggested that contact with industrial equipment, pipelines, storage surfaces, lubricants, auxiliary materials, or even the production environment may introduce trace amounts of BPA into the final product [45,59]. Because BPA is generally detected at extremely low concentrations, even minor external contamination sources may become analytically significant and contribute to the variability observed among studies.

Similarly, analytical contamination represents one of the major methodological challenges in BPA determination. The ubiquitous presence of BPA in the environment increases the risk of contamination during sample preparation and instrumental analysis [60,61,62]. Potential BPA contamination has been documented in laboratory solvents, filters, sample containers, pipette tips, and other consumables used throughout the analytical procedure. Consequently, the absence of appropriate procedural blanks and quality control measures may result in false-positive findings or overestimation of BPA concentrations. This issue may partially explain the discrepancies reported among studies employing different analytical protocols.

In addition, some authors have proposed that compounds generated during polymer degradation or naturally present in food matrices may interfere with BPA determination, particularly when less specific analytical methods are employed [63]. Under these circumstances, substances structurally related to BPA may be incorrectly identified as the target compound, thereby increasing the variability of published results. Although the development of mass spectrometry-based analytical techniques has substantially improved analytical selectivity and reliability, the possibility of analytical interferences cannot be completely excluded.

Overall, the available scientific evidence indicates that BPA in PET-packaged beverages should be interpreted from a multifactorial perspective that considers the packaging system as a whole [52,64,65,66]. To date, the literature does not conclusively demonstrate that PET itself is an intrinsic source of BPA. Instead, most evidence points to external contamination sources, cross-contamination during recycling and manufacturing processes, secondary packaging components, and methodological limitations associated with analytical determination. These factors largely explain the apparent inconsistencies among published studies and highlight the need for standardized experimental protocols, rigorous quality control procedures, and highly sensitive analytical methodologies to better clarify the true origin of BPA detected in beverages packaged in PET.

### 3.4. Trends and Future Perspectives in Research on BPA Migration from PET Packaging

**Figure 6:**
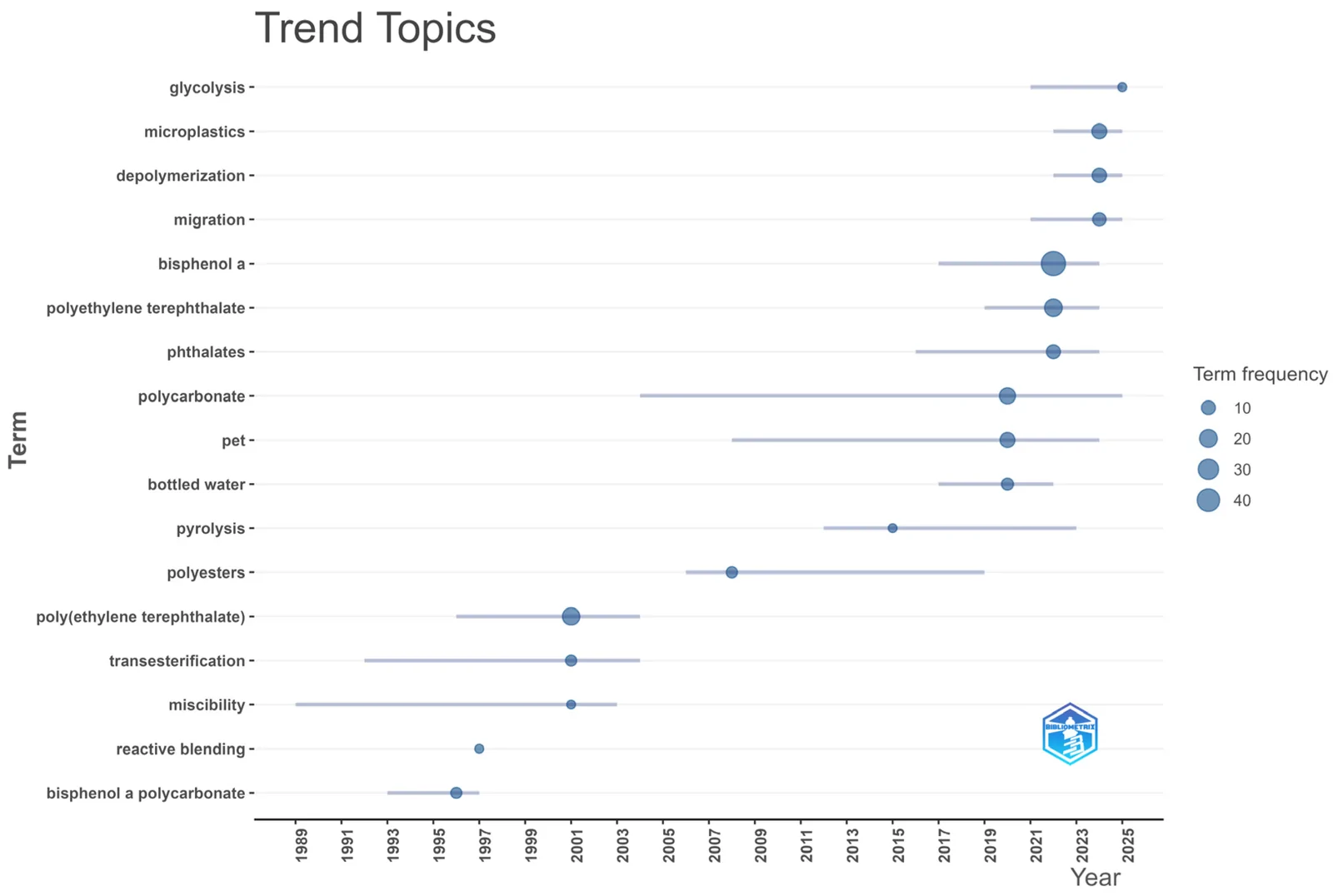
Trend topics and term frequency by year in the analyzed scientific literature.

The thematic trend analysis performed using the Bibliometrix package in RStudio (Figure 6) revealed a clear evolution in the scientific focus of research on BPA and PET over the past three decades. During the 1990s and the early 2000s, research primarily focused on the chemical properties and processing of polymeric materials, with keywords such as bisphenol A polycarbonate, reactive blending, miscibility, transesterification, and poly(ethylene terephthalate) being predominant. Between approximately 2005 and 2015, scientific interest gradually shifted toward polymer degradation and characterization, as reflected by the emergence of keywords such as polyesters, pyrolysis, PET, and polycarbonate. More recently, particularly since 2018, research has increasingly focused on food safety and public health, with bisphenol A becoming the most frequently occurring term and being closely associated with migration, bottled water, polyethylene terephthalate, and phthalates. Furthermore, the emergence of terms such as depolymerization and microplastics in recent years highlights growing interest in plastic recycling, the circular economy, and the potential release of contaminants from recycled polymeric materials. Overall, these findings indicate a transition from research centered on polymer chemistry to studies addressing contaminant migration, human exposure, and environmental sustainability.

Based on the findings of the present review, several priorities for future research were identified.

a) There is a need to develop and standardize analytical methodologies with greater sensitivity to accurately quantify BPA migration at trace levels under realistic storage and consumption conditions, particularly considering the increasing use of recycled PET (rPET).
b) Further studies should comprehensively evaluate the combined effects of environmental factors, including temperature fluctuations, ultraviolet (UV) radiation, repeated container use, and prolonged storage periods, on BPA migration into different food matrices. Future research should also expand its scope to include other bisphenol analogs (BPS, BPF, and BPAF), phthalates, microplastics, and other emerging contaminants that may simultaneously migrate from food packaging materials, thereby enabling cumulative exposure assessments.
c) The incorporation of probabilistic risk assessment models considering different population groups, consumption patterns, and chronic exposure scenarios is recommended to provide more realistic estimates of human health risks and generate scientific evidence that supports the development and strengthening of regulations governing food contact materials.

## 4. Conclusions

The scientific evidence analyzed confirms that the presence of BPA in beverages packaged in PET is a complex, multifactorial phenomenon. Although numerous studies have detected BPA in different PET-packaged food matrices, the reviewed literature does not conclusively demonstrate that this polymer is an intrinsic source of the contaminant. Instead, BPA migration is influenced by interactions among several factors, including temperature, storage time, ultraviolet radiation exposure, characteristics of the food matrix, packaging system composition, and methodological differences across analytical procedures. Furthermore, the available evidence indicates that sources such as recycled PET, secondary packaging components, cross-contamination during manufacturing, and potential analytical interferences are plausible explanations for BPA detection in PET-packaged beverages. Considering the potential of BPA as an endocrine-disrupting compound and the growing concern regarding chronic exposure at low concentrations, strengthening the standardization of analytical methods and conducting further research to clarify the actual source of this contaminant are essential. These efforts would contribute to a more accurate risk assessment and support improvements in regulatory strategies to ensure the safety of food-contact materials.

## Figures and Tables

**Table 1:** Occurrence and migration of bisphenol A (BPA) in beverages, with emphasis on PET packaging.

Sample Matrix	Evaluated Conditions	BPA Concentration (µg/L)	Analytical Method	LOD–LOQ (µg/L)	Main Finding	Ref.
Bottled water from two commercial brands	25–45 °C; 1–2 weeks	25.71–61.09	HPLC-FLD	0.15–0.48	BPA concentration increased with temperature; one brand exceeded the EU specific migration limit.	[26]
Bottled drinking water from five commercial brands (Jeddah, Saudi Arabia)	New PET bottles (200 mL); 23–25 °C, 40–43 °C (solar radiation), and 100 °C (30 min)	9.46–20.20	HPLC-DAD	Nr	BPA was detected in all samples. Migration increased significantly following solar exposure and thermal stress. Differences among brands suggested variations in PET quality. Estimated exposure remained below traditional safety limits but exceeded the most recent EFSA tolerable daily intake.	[44]
Commercial bottled drinking water (21 brands) and bottled mineral water (6 brands)	Commercial PET bottles; baseline storage and direct sunlight exposure for 10, 20, and 30 days (27.4–37.4 °C)	<LOD	HPLC-MS/MS	0.03–0.07	BPA was not detected in any sample, even after prolonged exposure to sunlight. The authors attributed the absence of migration to the use of high-quality virgin PET and suggested that BPA reported elsewhere may originate from recycled PET (rPET) or packaging components other than the bottle body.	[45]
Bottled water from five commercial brands (A–E) in Bauchi State, Nigeria	New PET bottles; protected storage and sunlight exposure for 14 and 28 days (47–56 °C)	<LOD (Day 0); 3.46–15.48	HPLC	Nr	BPA was not detected initially but increased progressively during storage, particularly under sunlight exposure. Temperature and solar radiation significantly enhanced migration, reaching a maximum of 15.48 µg/L.	[46]
Simulated purified water (Milli-Q, pH 6.0) in PET bottles from 16 commercial brands (Nanjing, China)	PET bottles refilled with Milli-Q water; 4, 25, and 70 °C for 1–4 weeks	0.00026–0.119	HPLC	0.23	BPA migration increased as a function of temperature and storage time, reaching 119 ng/L at 70 °C after 4 weeks. A positive correlation was also observed between bottle thickness, bottle mass, and initial BPA release.	[22]
Bottled drinking water (Poznań, Poland)	New PET bottles (500 mL); incubation for 24 h at 8, 18, 28, 38, and 48 °C.	0.00163–0.01854	LC-MS/MS	0.23–Nr	BPA migration exhibited a non-linear thermal behavior, increasing up to 28 °C, decreasing at 38 °C, and rising again at 48 °C. Concentrations remained below the EU specific migration limit.	[47]
Bottled drinking water (Selangor, Malaysia)	Comparative evaluation of PET and polycarbonate (PC) bottles under ambient storage, elevated temperature, and sunlight exposure inside vehicles	0.01153–0.26987	UHPLC-FLD	Nr	Contrary to expectations, PET bottles showed higher BPA concentrations than PC bottles under all evaluated conditions. Elevated temperature, prolonged storage, and solar exposure promoted migration, with the highest levels found in bottles stored inside sun-exposed vehicles. Non-carcinogenic risk remained low (HQ < 1).	[48]
Commercial bottled water (Guangzhou, China)	Unopened commercial samples stored at room temperature; mineral, natural drinking, and purified water	PET: 0.0124–0.0449	GC-MS SIM.	0.00068–0.00227	BPA was the predominant bisphenol detected. Polycarbonate containers exhibited average BPA concentrations approximately 67 times higher than those of PET bottles, representing a substantially greater source of consumer exposure.	[49]
Mint-flavored yogurt drink (Doogh)	New PET containers; refrigerated (4 °C), room temperature (25 °C), heated (45 °C), prolonged storage (7–60 days), and 30-day sunlight exposure	107–340	HPLC-UV	90–280	Elevated temperature, prolonged storage, and direct sunlight significantly increased BPA migration, reaching the highest concentration (0.340 mg/L) under direct solar exposure. Differences among brands suggest an influence of packaging formulation or polymer characteristics.	[14]
Commercial kefir	Ready-to-drink commercial products purchased from the market; baseline BPA determination	4.65–5.73	GC-MS/MS.	0.01	BPA was detected in 100% of PET-packaged kefir samples. Although concentrations complied with the EU specific migration limit, estimated exposure suggested potential health concerns because the tolerable daily intake proposed by EFSA could be exceeded with continuous consumption.	[50]
Carbonated beverages: canned, glass-bottled, and PET-bottled	Different flavors, origins, and packaging materials; 250–1000 mL.	0.37–21.83	SPE–UPLC-MS/MS	0.02–0.04	BPA was detected in PET-bottled beverages at concentrations of 0.37–21.83 µg/L, confirming its presence in PET-bottled beverages.	[51]
Beer packaged in PET bottles	4 and 20 °C; 0–4 months of storage; 2 L PET bottles	0.020–highest value at 20 °C after 4 months	GC-MS	Nr	BPA concentration increased with storage time and temperature, with the highest observed after 4 months at 20 °C, indicating greater BPA migration from PET during prolonged storage.	[52]
Fruit juice (PET, multi-layer PPA, and glass)	Different packaging materials, juice types, and origins; commercially available samples.	PET: 0.57–28.97; multi-layer PPA: ND–5.48; glass: ND–0.43	SPE–UPLC-MS/MS	0.04–0.13	BPA was detected in approximately 83% of fruit juice samples. PET-packaged juices showed the highest BPA concentrations (0.57–28.97 µg/L), indicating a higher prevalence of BPA in beverages packaged in PET than in multi-layer PPA and glass.	[53]
Food simulants: Simulant A (10% ethanol) and Simulant B (3% acetic acid)	New PET containers; ultrasound treatment (20 kHz, pulse mode, 10–30 min), storage at 25–45 °C for 1–2 weeks, and UV-C exposure (200–280 nm, 1–6 h)	1.36–4.00	HPLC-FLD	0.15–0.48	Ultrasound treatment and UV-C radiation promoted the greatest increases in BPA migration, exceeding the effects observed under conventional thermal storage conditions.	[26]

Note: BPA concentrations originally expressed in ng/L or mg/L were converted to µg/L using standard SI conversion factors (1 mg/L = 1000 µg/L; 1 µg/L = 1000 ng/L) to facilitate comparison across studies, as current migration limits and regulatory assessments are commonly expressed in µg/L. Nr: not reported.

## Data Availability

No new data were created or analyzed in this study. Data sharing is not applicable to this article.

## Supplementary Materials

The following supporting information can be downloaded at: https://www.mdpi.com/article/10.3390/toxics14090796/s1, Figure S1: Most Relevant Authors; Figure S2: Most Relevant Affiliations.
