# Supplementary material for: Migration of Bisphenol A (BPA) into PET-Bottled Beverages: A Comprehensive Review and Bibliometric Analysis
Source: Toxics. 2026 Sep 9;14(9):796. doi: 10.3390/toxics14090796 (PMC13611502; doi:10.3390/toxics14090796)
Supplement: Supplementary file 1 [file toxics-14-00796-s001.zip › toxics-4481223-supplementary.pdf]

Supplementary material

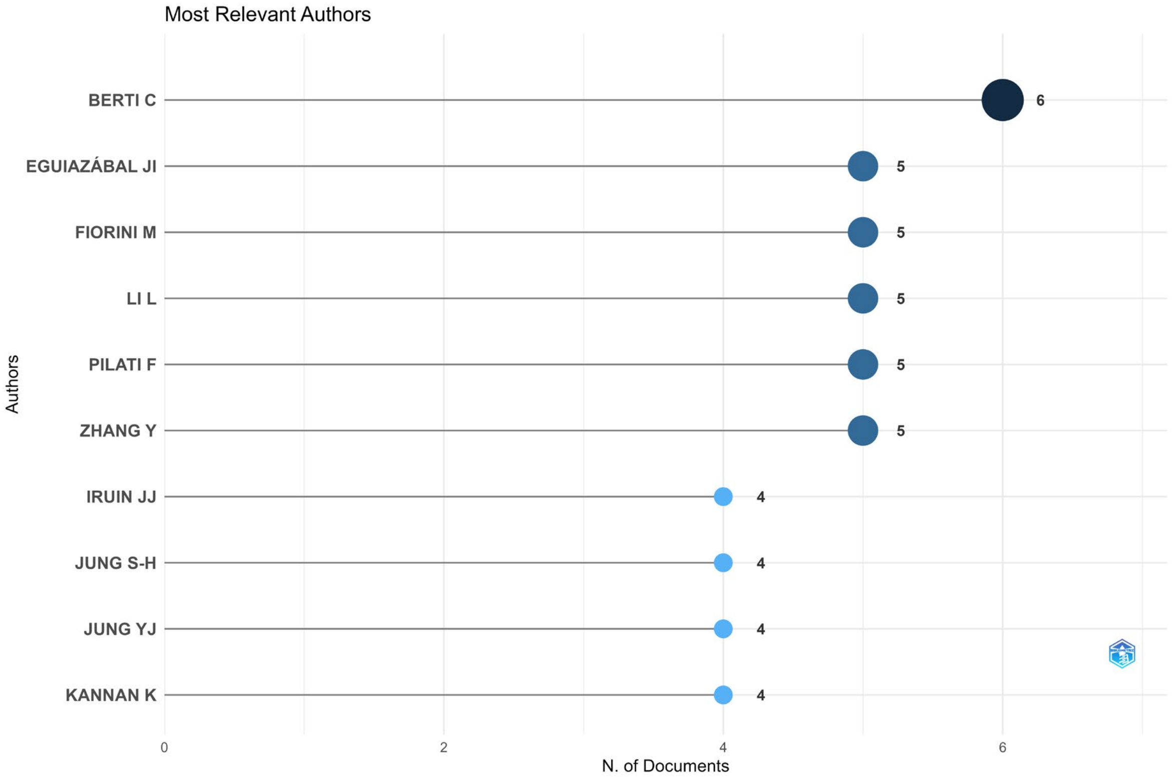

Figure S1. Most Relevant Authors.

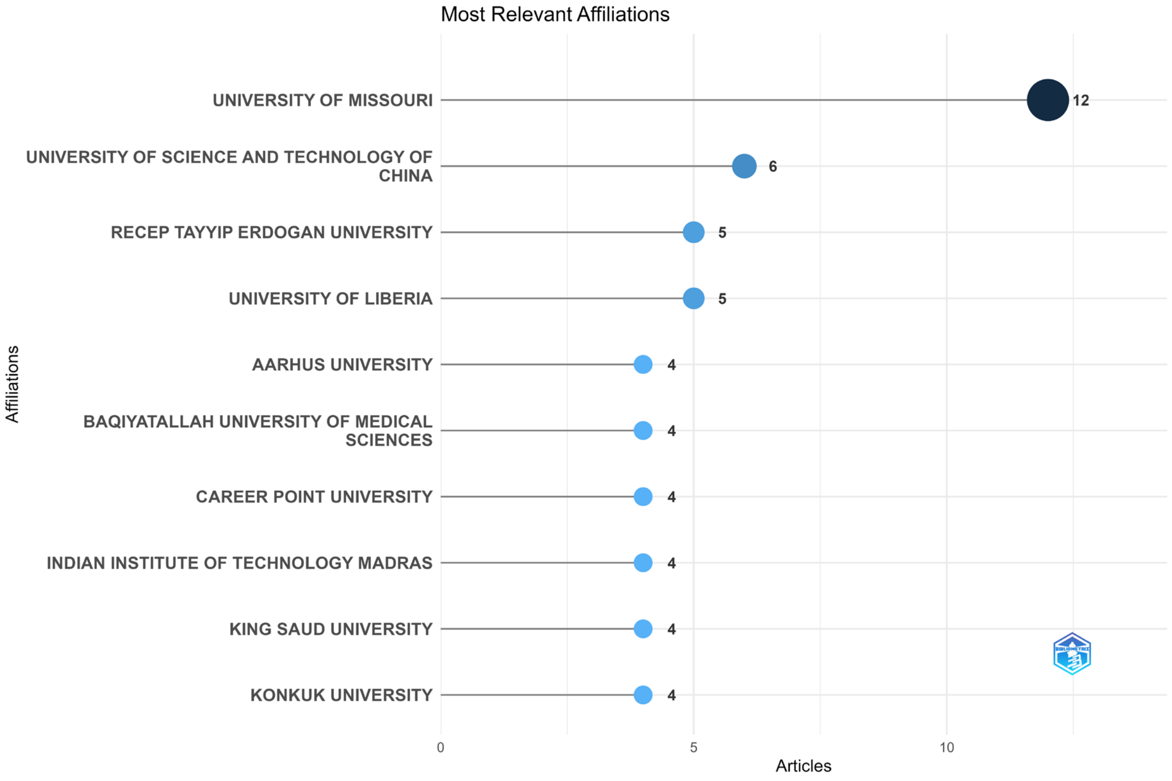

Figure S2. Most Relevant Affiliations.
